# Cephalometric evaluation of the oropharyngeal space in children with atypical deglutition

**DOI:** 10.1590/S1808-86942012000100019

**Published:** 2015-10-20

**Authors:** Almiro José Machado Júnior, Agrício N. Crespo

**Affiliations:** a DDS, Specialist in maxillary functional orthopedics; MSc in Medical Sciences - Unicamp (PhD student - School of Medical Sciences - University of Campinas - Unicamp); b PhD (Head of the ENT program - Unicamp). FMC - Unicamp. Capes.

**Keywords:** deglutition, oropharynx, radiography

## Abstract

For several factors, not yet fully explained until now, infant deglutition may persist after changing the primary teeth and such swallowing is classified as atypical swallowing. Possible causes: finger sucking, bottle feeding, sucking the tongue and mouth breathing. There is no consensus about the etiology of atypical deglutition.

**Objective:**

The aim of this study was to compare the oropharyngeal space in side-view radiographs of children with atypical deglutition and normal deglutition.

**Methods:**

Retrospective study, by means of cephalometric analysis of side-view radiographs, measuring the anteroposterior distance of the lumen of the airway in two groups: 55 cephalograms from the experimental group (with atypical deglutition) and 55 side-view radiographs from the control group (normal deglutition). Measurements from the groups were compared using Mann-Whitney U test and a *p* value <0.05 was considered as an indication of statistical significance.

**Results:**

The median in the control group was 10 mm and in the experimental group it was 7 mm, with a statistically significant difference (*p* <0.001).

**Conclusion:**

The oropharyngeal space is reduced in the group with atypical deglutition.

## INTRODUCTION

Three swallowing patterns have been described: visceral, somatic and irregular. Visceral is the swallowing at birth, also known as infantile swallowing. The tongue is relatively large in newborns and placed more anteriorly, between the anterior gum rollers and it aids in anterior lip sealing^1^, ^2^, ^3^, ^4^, ^5^. With the eruption of the first deciduous teeth, the suction push reduces and is slowly replaced by the push to bite and, at this stage; deglutition is called irregular^1^, ^2^. Although deglutition is the first function established in the stomatognathic system, it is the last process to mature, because as bone structures are under growth and dentition is still not installed, the tongue cannot acquire mature posture and movement. It is only when the child is about two years of age, a transitional (irregular) deglutition pattern is expected for the mature pattern, with the tongue within the confines of the teeth arches, with the soft tissue better adjusted and the lips sealed, called somatic deglutition^6^, ^7^, ^8^.

Mixed dentition stage is a phase of development in which there are numerous changes to the stomatognathic system, and studies say that it is at this stage that there should be the definitive transition from the infantile deglutition pattern (visceral) to the mature pattern (somatic). For a number of reasons which are still incompletely explained, “infantile deglutition” may continue to beyond the replacement of the deciduous teeth, being classified as atypical deglutition^3^, ^5^, ^8^. Atypical deglutition has been attributed to suction without nutritional purposes, use of bottles, oral breathing, central nervous system disorders and anatomical changes^5^, ^6^, ^7^, ^8^, ^9^. Notwithstanding, there is no consensus regarding its etiology.

Studies have shown that deglutition is an activity which is coordinated together with other oral functions, and it requires a straight interaction between different muscle groups. In order to synchronize suction and swallowing, it is necessary to have a very close relationship between the muscles in the oral region in the generation of suction pressure - to open and close the mouth, and the tongue in order to form the bolus and its peristaltic transportation to the pharynx^10^. During oral feeding, respiratory mechanics involves the proper activation of the diaphragm, intercostal muscles and the upper airway muscles – from the nose to the glottis^10^. Recent studies have reported that adenoid and palatine tonsil hyperplasia is the second most frequent cause of upper airway obstruction and, consequently, oral breathing in children^11^, ^12^, ^13^, ^14^, ^15^. The relationship between oral breathing and atypical deglutition has already been studied^16^, ^17^, ^18^, ^19^, ^20^, but it is still controversial^21^. One recent study^22^ assessed the oropharyngeal distance in teleradiography made in the orthostatic position; nonetheless, it did not detect differences between the groups studied and in the different age ranges evaluated. Therefore, the goal of the present study was to compare the oropharyngeal distances in teleradiographies, taken in natural head position of children in physiologically mixed dentition stage, with atypical deglutition and normal deglutition.

## MATERIALS AND METHODS

**Figure 1:**
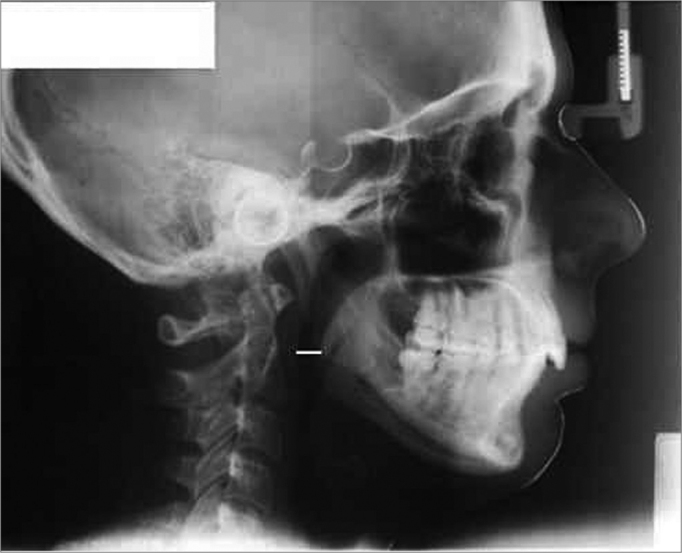
PAS (lower airway) Cephalometric value: pharynx width at the point where, radiographically, the posterior tongue border crosses the inferior mandible border, all the way to the closest point to the posterior pharyngeal wall.

In this retrospective, analytical and observational cross-sectional cohort, we assessed side-view teleradiographies from children of both genders, in mixed physiological dentition, between 7 and 11 years of age^1^, ^2^, ^3^, ^5^, treated in the functional orthopedics of maxillae program of the Systemic Dentistry Society of São Paulo Clinic. All side-view teleradiographies selected had the following characteristics: 18x24 cm, taken with a Siemens device, for 1 second, 6 Kvp and 1.5 meters of focal distance, carried out with the patient under natural head position. After selecting the side-view teleradiographies, we carried out a cephalometric exam in a dark room, using a radiologic view box, overlapping an acetate sheet over the teleradiography. We outlined the anatomo-radiographic structures of the PAS variable, also called lower airway: pharyngeal width where, radiographically, the posterior tongue border crosses the lower mandible border, all the way to the point nearest the posterior pharyngeal wall^20^, ^23^(Figure 1).

Side-view radiographs without good visibility of anatomical structures used in the cephalograms were taken off the sample. Patients with dental agenesis; congenital orofacial malformations; orthodontic, functional orthopedic and orofacial myofunctional treatments prior to the study, doubts or inaccuracies as to the swallowing diagnosis were taken off the study. In order to define the control and experimental groups, we used the labial opening pressure test with saliva swallowing^2^, ^3^, carried out by three functional orthopedists/orthodontists simultaneously, settling, by consensus, to which group the child's teleradiography would belong to:

- Normal deglutition (control group): labial sealing and positioning the tongue tip on the papila^21^;
- Atypical deglutition (experimental group): atypical labial pressure, tongue pressure against the anterior teeth or between the dental arches^21^.

We selected 20 side-view teleradiographies from 20 patients diagnosed with atypical swallowing and 20 side-view teleradiographies from 20 subjects clinically diagnosed with normal swallowing. With these teleradiographies, we carried out a pilot study in order to calculate the size of the sample: we calculated the control group's standard deviation and the difference between the mean values from the control and experimental groups; with a significance level of 0.05 and test power of 0.10, we obtained the desired sample size of 110 teleradiographies, 55 in each group. After we calculated the sample size, we selected the sample based on the same criteria of the pilot study aforementioned.

The side-view teleradiographies of the study and control groups were randomly classified and numbered in a sequence. This procedure was carried out so that the examiner, who did the measurements manually, would be blind as to which group the teleradiography belong to, thus avoiding biases. The numbered teleradiographies were handed over to the examiner in order to carry out the aforementioned standardized measures, writing them down in the data collection tool. In order to minimize systematic error, the same examiner collected the data from the entire sample at two occasions, within a 20-day interval between them.

After measuring and writing down the data corresponding to all the radiographs, information regarding age, gender and the presence or absence of atypical deglutition was added. In order to compare the variables between the two groups, we used the Mann-Whitney, test, finding the mean, median, minimum median, maximum median, standard deviation and the test values assessing data significance. In order to control variables age and gender, we carried out a covariance analysis (ancova). In order to check for intra-examiner consistence, we employed the Wilcoxon test for related samples, on a possible difference of measures at the two data collection times. The significance level used for the statistical tests was 5%.

Because this is a retrospective study using side-view teleradiographies from patients who have been already treated, and also because this study did not do experiments with human beings, it did not require an informed consent form, making sure that all necessary measures were taken in order to maintain the confidentiality of the information given by the patients. Only the patients initials were written down into the collection device, and there was no way that any other person, except for the researcher, could identify to whom each teleradiography belonged. The study protocol for this study was previously approved, without restrictions, by the Ethics in Research Committee of the Institution.

## RESULTS

This study's sample involved 110 side-view teleradiographies, belonging to 52 female patients and 58 males, and such gender difference was not significant (*p*=0.1266). The mean age of the control group (normal deglutition) was 9.46 years (standard deviation = 1.83) and 10.05 years in the experimental group (standard deviation = 2.13), without significant difference (*p*=0.6345).

**Figure 2:**
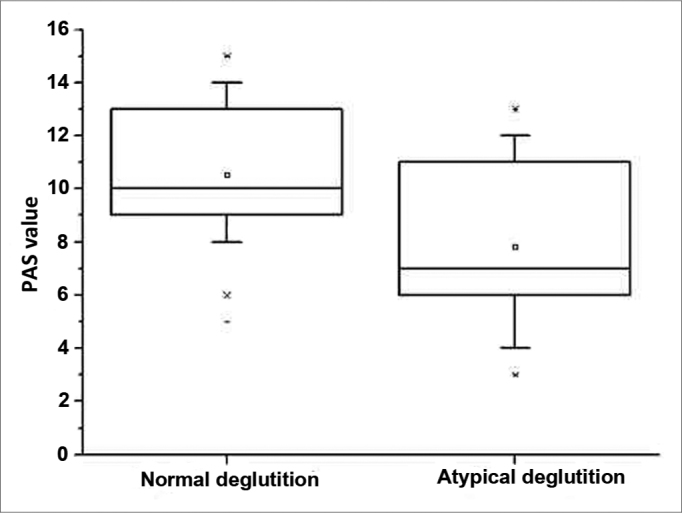
Set of PAS value data from the normal and atypical deglutition groups. The inner line of the box outlines the data set median value.

In order to compare the values between the two groups we used the Mann-Whitney test. The median width of the PAS variable was 7 mm, in the experimental group and 10 mm in the control group (Table 1 and Figure 2), with a statistically significant difference (*p* <0.001). This significance was maintained after employing the covariance analysis (ancova) in order to control the age and gender variables in the samples. To assess intra-examiner uniformity, we employed the Wilcoxon test for selected samples on the possible difference of the measures at the two data collection occasions, and we did not find statistically significant differences concerning this possible difference (*p*=0.989).

**Table 1:** Comparative analysis of the PAS variable in millimeters.

swallowing	n	mean	Standard deviation	minimum	median	maximum	Mann-Whitney *p*-value
normal	55	10.53	2.43	5.00	10.00	15.00	<0.0001*
atypical	55	7.82	2.93	3.00	7.00	13.00	

* There was a significant difference: *p*-value < 0.05.

## DISCUSSION

This study's results showed a significant difference in the PAS radiographic measure between the groups studied. The value found (10mm median) in the control group is very similar to the value indicating normality^20^. The age at which a child reaches the mature deglutition standard is controversial in the literature, varying between 18 months and 6 years of age^21^. Therefore, we chose to collect the data from children at the age of mixed physiological dentition (between 7 and 11 years)^1^, ^3^, ^4^, ^5^.

Although the age range investigated is broad, the results from this study did not show statistical differences between groups with regards to age of the subjects. Still, assuming that age and gender could be variables which could increase or decrease of the value studied, we analyzed the covariance (ANCOVA) in order to control age and gender variables. This threshold showed that there still is a significant difference (*p*<0.001) between the two groups for the PAS variable, regardless of age and gender.

One study^21^ associates atypical deglutition and occlusal problems in oral breathing children and did not find a statistically significant correlation between atypical deglutition and anterior open bite in 8 to 12 year-old oral breathers. The authors also stated that dental occlusion is not responsible for these children having inadequate deglutition, it is rather associated with the open mouth posture they have in order to keep the air flow and/or because of a hyperplastic palatine tonsil. We believe that the results from the present study may support such hypothesis, since the airway reduction, very likely caused by tonsil hyperplasia, would trigger the oral breather syndrome, change in tongue posture and, consequently, causing atypical deglutition.

PAS has been described as the distance between the back of the pharynx to the front, where the tongue base is^20^. We believe that factors such as age and gender are perhaps less important than the posture of the tongue in the allocation of the PAS variable radiographic value. Perhaps, it is the tongue posture that defines this distance, and not the airway lumen itself.

Ceylan & Oktay^10^ noticed that the radiographic anatomy of the pharyngeal space is related to the mandibular position. We believe that the lowered tongue may be able to keep the mandible in a posterior position and trigger distal occlusion, a frequent type of malocclusion in oral breathers^22^.

Vieira & Villela^22^ performed cephalometric studies evaluating oropharyngeal distance values in atypical swallowing in different age groups in radiographies taken in the standing position. A difference of the present study is the use of teleradiographies taken with the patient in natural head position. We believe that the change in head tilt in the radiograph taken with the patient standing, may change the airway measurement; nonetheless, further studies are needed in order to assess this issue. However, as for the age data, the results of this study agree with the findings from Vieira & Villela^21^, because the value studied did not vary with age.

We collected data from the entire sample at two times in an attempt to minimize a systematic error. We checked to see whether the data collected by the same examiner at two different occasions could vary significantly. This was not the case - proving an intra-examiner consistency. Although the variables are measurable, we used a nonparametric statistical test, due to abnormal sample data distribution. Since this study was retrospective, based on teleradiography analyses, one limitation was that it was not possible to assess whether the distance measured can be changed after correction of the swallowing movement disorder. Further studies should be conducted to evaluate this hypothesis.

Despite the focus given to the dental study, we believe that this is a multidisciplinary subject: orthodontics, functional orthopedic, pediatric dentistry, ENT, pediatric and speech therapy. So much so that various areas of medicine have studied possible craniofacial changes in children with respiratory obstruction^11^, ^12^, ^13^, ^14^, ^15^. The absence of a direct relationship between the cause of airway obstruction and its effect on craniofacial growth^11^, ^12^, ^13^, ^14^, ^15^ leads to a considerable controversy in the literature. The most accepted theory is that tonsil hyperplasia leads to pharyngeal obstruction, causing mouth breathing, and changes to the position of orofacial and mandible muscles^11^. These changes, in turn, influence chewing, swallowing and speech, and lead to occlusal and skeletal changes.^19^, ^20^, ^21^ The results of this study show that in the group of atypical swallowing, there is airway reduction. Perhaps, such reduction is caused by hyperplasia of the palatine tonsil, leading to pharyngeal obstruction, causing mouth breathing. Data from this study may corroborate the hypothesis of the relationship between mouth breathing and atypical swallowing^21^. We suggest further studies to evaluate this hypothesis.

Authors have suggested oral breathing as an etiologic factor in atypical swallowing^18^, ^19^, ^20^, ^21^. The results from this study do not enable us to answer to this hypothesis. However, we believe that reducing the oropharyngeal space, as assessed by PAS in this study, is the main cause of both: mouth breathing and atypical swallowing. We believe that atypical deglutition and mouth breathing should be evaluated and treated together. We also believe that these disorders are part of a more complex picture, in which atypical swallowing and mouth breathing are just part of the problem.

## CONCLUSIONS

- The cephalometric analysis of the PAS revealed a mean difference of about 3 mm between the two groups, with statistical significance;
- The PAS distance, based on radiographic measurements, was lower in children with atypical swallowing.
